# Determinants of Outpatient Physician Visits and Hospitalizations in Bulgaria: A Cross‐Sectional Study

**DOI:** 10.1002/hsr2.70800

**Published:** 2025-07-30

**Authors:** Svetlana Panayotova, Elka Atanasova

**Affiliations:** ^1^ Department of Health Economics and Management, Faculty of Public Health Medical University – Varna Varna Bulgaria

**Keywords:** Andersen's behavioral model, Bulgaria, determinants of outpatient physician visits, health care use, health care utilization, hospitalizations

## Abstract

**Background and Aims:**

The aim of this study was to identify the determinants of outpatient physician visits and hospitalizations in Bulgaria. Given resource disparities and hospital overutilization, understanding healthcare misuse or underuse could improve system efficiency and cost management.

**Methods:**

An online survey was conducted in Bulgaria (January–February 2023) among individuals aged 18+ to collect information on individual characteristics and the use of health services. The cross‐sectional study analyzed primary data from 1292 respondents. Determinants were selected based on the Andersen's behavioral model. The number of outpatient physician visits (GP, specialist) as well as the number of hospitalizations in the past 12 months were used as outcome measures. Four negative binomial regressions were conducted to assess associations between the independent and the dependent variables.

**Results:**

GP visits were positively associated with family size and trust in provider. Older age, higher education, and part‐time or unemployed status were associated with lower private visits to specialists. Higher income predicted fewer GP and referral‐based specialist visits. In contrast, long waiting times, distance, and transportation challenges increased private consultations. Better self‐rated health was a negative predictor of GP and specialist visits. The presence of one or more chronic conditions was associated with more outpatient physician visits and more hospitalizations.

**Conclusion:**

We have applied the Andersen's behavioral model, facilitating comparative analyses based on results in different countries and health systems. Future studies should explore how these utilization patterns influence health outcomes in Bulgaria.

## Summary

- Identification of the determinants of outpatient physician visits and hospitalizations in Bulgaria based on Andersen's behavioral model of Health Services Use.
- A prerequisite for comparative analyses in different countries and health systems.
- Identification of the impact of utilization rates on population health is needed.

## 1 Background

Knowledge about health services utilization determinants is critical to the identification of reasons behind differences in access, customer satisfaction, and health outcomes. Moreover, understanding the factors that influence health care services utilization provides an opportunity to predict and manage the use of health services and associated costs.

While several studies examined different factors affecting the use of health care services in Bulgaria, there is a general lack of studies applying a particular model for health care utilization and identifying the determinants that best predict healthcare use in outpatient and inpatient care in the country. A literature review revealed that Ronald Andersen's behavioral model of Health Services Use (hereinafter referred to as the Behavioral Model or Andersen Model) was one of the most commonly engaged models in health service utilization research [1, 2]. According to Andersen model, the use of health care services is a function of three components of individual characteristics: predisposing characteristics (individual's predisposition to use such services), enabling resources (factors that enable or impede the use of services), and need factors (need for health services) [3]. Since the Behavioral model has been used in many studies on the determinants of health care use, it was chosen for this study [2, 4, 5].

A brief review of some of the important aspects of the Bulgarian healthcare system can help the reader better understand the findings of the present research. A health insurance system, with compulsory and voluntary health insurance, was established by the 1998 Health Insurance Act. Approximately 88% of the population was covered by statutory health insurance (SHI) and around 12% of the population did not have health insurance in 2020 [6]. The SHI provides a basic package of health care services through the budget of the National Health Insurance Fund (NHIF), which is the single public payer. Voluntary health insurance (VHI) has never played a significant role in the insurance model. General practitioners (GPs) are independent practitioners, operating mostly in individual or less often in group practices, and are gatekeepers to publicly financed specialized care. Hospital care is delivered by public and private providers and it is available with a referral from an outpatient physician or in an emergency.

In addition, Bulgaria has a mixed public–private health care financing system. In 2021, Bulgaria spent 8.6% of its gross domestic product on health, where public sources represent 65% of spending [7]. Out‐of‐pocket payments account for 34% of private spending and are mostly related to prescription drugs and direct payments for services that are not covered by the NHIF benefits package. These payments are the highest in the European Union where the average is 15% [8]. Dimova et al. provided additional information about the Bulgarian health system [9].

Unbalanced distribution of resources (human and physical), shortage of GPs and nurses, dominance of the hospital sector are among the main issues in the Bulgarian healthcare context. Overutilization of hospital care in Bulgaria does not lead to optimal outcomes in terms of population health indicators [10]. In sum, knowledge about a potential overuse or underuse of health care services may help to improve the organization and manage the economic burden on the healthcare system. For this reason, it is important to clarify the determinants of outpatient physician visits and hospitalization. Hence, the aim of the present study is to explore the association between the determinants and the quantity of health care services used in outpatient and inpatient care in Bulgaria. Andersen's model serves as the study's framework. Furthermore, we demonstrate the relevance of the Behavioral model in the Bulgarian context.

## 2 Methods

A cross‐sectional study was carried out to analyze the influence of individual characteristics on outpatient physician visits and hospitalizations in Bulgaria.

### 2.1 Sample

A cross‐sectional online survey was conducted in January–February 2023 in Bulgaria. The target population included individuals aged 18 years and older who had lived in the country for the past 12 months. A total of 1748 people agreed to participate in the survey. After listwise deletion of missing values, the final sample included 1292 participants with complete data (74% response rate). Respondents were recruited nationwide using the snowball sampling method. An invitation to complete the survey was distributed via email and shared on social networks and groups across all six regions of the country. Participants completed a standardized online questionnaire, accessible via an online platform. The online survey enabled access to a wider range of respondents, reduced time and resource costs, and provided flexibility for respondents to complete the questionnaire at their convenience. All participants provided written informed consent to participate in the study before the beginning of the survey, which is a common procedure in online surveys. The study collected primary information on individual characteristics and use of health care services in the previous 12 months.

### 2.2 Outcomes (Dependent Variables)

The self‐reported number of visits to physicians and hospitalizations in the past 12 months were used as outcome measure:

- Number of GP visits.
- Number of visits to medical specialists with a referral under the SHI scheme.
- Number of visits to medical specialists in the private sector.
- Number of hospitalizations (planned and emergency hospital stays).

Respondents were asked to report how many times they had visited a doctor and how many times they had been admitted to a hospital in the past year.

### 2.3 Independent Variables

We defined individual characteristics as independent variables in the analysis, according to Andersen's conceptual framework. Predisposing, enabling, and need characteristics served as potential determinants of the number of used health care services.

*Predisposing factors* were age, gender, family status (never married; married or living with a partner without marriage; divorced or widowed), family size (number of persons in the family), place of residence (capital city, regional city, small town, and rural area), employment status (full‐time job; part‐time job or unemployed; retired due to age or disability), and educational level (International Standard Classification of Education [ISCED 2011]) [11]. To examine individual health beliefs, respondents were asked to rate how much they trust the doctor or hospital they visited. Their trust in health care providers was assessed using a five‐point Likert scale (from 1 = “not trusted at all” to 5 = “trusted very much”).

With regard to *enabling factors*, SHI status, VHI status, and monthly household income in Bulgarian Lev (BGN) were included. Respondents stated their SHI status (“I have continuous SHI”/“I have SHI with interruptions”/“I don't have SHI”) and whether they had VHI (yes or no). Moreover, “too expensive health care services,” “long waiting times or waiting lists,” “no time because of work and personal commitments,” “distance to travel and/or transportation difficulties,” and “lack of equipment and supplies at the doctor's office” were considered access barriers that represent enabling factors. The evaluation scale ranged from 1 (“This does not concern me at all”) to 5 (“totally applies to me”). For the purpose of the regression analysis, the variables were dichotomized into “applies to me” and “not applicable.”

*Need factors* covered self‐rated health status and the presence of a chronic disease. Respondents were asked to assess their health on a scale from 1 (“very bad”) to 5 (“very good”) and to indicate whether they have a chronic condition diagnosed by a physician (yes or no).

### 2.4 Statistical Analysis

Descriptive statistics were first performed to illustrate the survey outcomes, including the frequency distribution of the respondents and the mean values of the sample parameters.

Then, a multivariate regression analysis was conducted to investigate the influence of individual characteristics (multiple independent variables) on the use of health care services (outcome/dependent variable). The independent variables were represented by nominal, ordinal and interval data, and the dependent variables were represented by interval data—number of services used in the last 12 months (number of GP visits, number of specialist examinations with a referral, number of specialist private examinations, number of hospitalizations).

Since the data for the dependent variables did not meet the requirements for a normal distribution and a large variance (overdispersion) was observed, linear regression and Poisson regression were not appropriate methods to perform the planned analysis. Negative binomial regression is a type of Poisson model that is applied to data with a high variance [12]. This type of regression analysis is the statistical method in many studies, investigating the factors influencing health service utilization [13, 14, 15, 16, 17].

Four negative binomial regressions were conducted to examine the influence of individual characteristics on the number of health services used: GP check‐ups, specialist check‐ups with referral, private specialist check‐ups, and hospitalizations. Adjustments were made for predisposing characteristics, enabling resources, and need factors. The aim was to understand how much each of the factors affects the use of services, holding other variables constant.

We added the predisposing factors, enabling factors, and need factors into the regression model in a stepwise manner as independent variables, creating three models for each type of service:

− Model 1—includes predisposing characteristics;
− Model 2—includes predisposing and enabling characteristics;
− Model 3—includes predisposing, enabling, and need characteristics.

When additional sets of variables were added in the second and third models, some factors lost statistical significance, while for others, significance emerged in the full model.

The sequential introduction of each of the three components allows for the assessment of the additional influence of each group of factors on the utilization of the service. This sequence follows the structure of the behavioral model and established practices in the reviewed literature [14, 15].

Model goodness‐of‐fit was assessed using Pearson chi‐square and Akaike's information criterion (AIC), and model fit was evaluated using McFadden's pseudo *R*‐square. The full model (Model 3, including predisposing, enabling, and need characteristics) demonstrates the best fit to the data for all four outcome variables. Therefore, in Table 2, we report the results for the full regression models.

The regression analysis results identify the individual characteristics that significantly and independently affect the quantity of health care services used, the direction and the magnitude of their influence. Positive B‐coefficients indicate increased use, while negative B‐coefficients indicate decreased use as the independent variable increases (or in comparison to the reference group). The incidence rate ratio (IRR) measures the expected change in the number of health services used as a result of a one‐unit change in the independent variable (or compared to the reference group in nominal and ordinal data) [12].

The significance level was set at *p* < 0.05 (two‐sided). Specialized software (IBM SPSS Statistics, Version 19) was used for statistical processing and data analysis [18].

### 2.5 Ethical Issues

The collection and use of personal information before, during, and after the completion of the research is carried out in accordance with the General Data Protection Regulation (Regulation 2016/679) [19]. The Research Ethics Committee of the Medical University of Varna approved the study.

## 3 Results

### 3.1 Sample Characteristics

Sample characteristics are shown in Table 1. The majority of respondents are women (77.3%) and fall into the 25–44 age group. Of all respondents, 53.3% are married, while 21.3% live with a partner but are not married. The average household size in the sample is 2.92 (SD: 1.08), with two people (30.3%) or three people (29.9%) making up the majority of households. Most of respondents live in a regional city (67.6%), reported higher education level (master's degree—41.7%) and have a full‐time job (67%).

**Table 1:** Sample characteristics (*N* = 1292).

Variable	Description	Frequency (number)	Percentage
**Predisposing characteristics**				
*Age (mean/SD)*		44.94	13.59
Age group	18–24 years	86	6.7
	25–44 years	570	44.1
	45–64 years	516	39.9
	≥ 65 years	120	9.3
Gender	Male	293	22.7
	Female	999	77.3
Family status	Never married	178	13.8
	Married/living with a partner	964	74.6
	Divorced/widowed	150	11.6
*Family size (mean/SD)*		2.92	1.08
Place of residence	Capital (Sofia)	96	7.4
	Regional city	874	67.6
	Small town	196	15.2
	Rural area	126	9.8
Education level	Lower secondary or lower (ISCED 11 Level 0–2)	14	1.1
	Upper secondary or vocational (ISCED 11 Level 3–4)	403	31.2
	Professional bachelor degree (ISCED 11 Level 5)	80	6.2
	Bachelor degree (ISCED 11 Level 6)	217	16.8
	Master degree (ISCED 11 Level 7)	539	41.7
	Doctoral degree (ISCED 11 Level 8)	39	3.0
Employment status	Full‐time job	865	67.0
	Part‐time job/unemployed	269	20.8
	Retired (due to age or disability)	158	12.2
**Enabling characteristics**				
Statutory health insurance	Yes, continuous	1217	94.2
	No/with interruptions	75	5.8
Voluntary health insurance	Yes	281	21.7
Household income	Under BGN 700	61	4.7
	BGN 701–1200	191	14.8
	BGN 1201–2000	362	28.0
	BGN 2001–3400	322	24.9
	Over BGN 3400	143	11.1
	Do not want to answer	213	16.5
**Need characteristics**				
Self‐rated health	Very bad	12	0.9
	Bad	83	6.4
	Satisfactory	425	32.9
	Good	546	42.3
	Very good	226	17.5
Chronic disease	Yes	565	43.7

Abbreviations: BGN, Bulgarian Lev; ISCED, International Standard Classification of Education; *N*, total sample size; SD, standard deviation.

Regarding the trust in GP's, specialist doctors and hospitals, we find that the specialists have the highest average trust score—4.11, followed by GPs—3.92 and hospitals—3.31 on a five‐point scale. The largest proportion of respondents have a household income of BGN 1201–2000 (28%). The vast majority of the participants (94.2%) indicated that they have been covered by SHI in the past 12 months, and 21.7% have voluntary health insurance coverage funded by an employer or by themselves.

The majority of respondents rated their health as good (42.3%) or fair (32.9%). Nearly half of the study participants (43.7%) admitted to having one or more chronic conditions, where cardiovascular diseases prevail (18.3% of all respondents, or 41.9% of those with chronic illnesses). Further details are shown in Table 1.

### 3.2 Use of Health Services

Among 1139 respondents who had seen a GP in the past 12 months, the average number of GP visits was 4.14 (95% confidence interval [CI]: 3.94–4.34; SD: 3.42). Average specialist visits with a referral in the past 12 months equaled 2.79 visits (*N* = 908; 95% CI: 2.65–2.93; SD: 2.17) and average private specialist visits equaled 2.51 (*N* = 651; 95% CI: 2.34–2.68; SD: 2.22). For the 253 respondents who were admitted to the hospital in the last year, the average number of hospitalizations was 1.64 (95% CI: 1.47–1.82; SD: 1.45).

### 3.3 Regression Analysis

Results of multiple negative binomial regression analysis are presented in Table 2. Data show the individual characteristics that are associated with the number of GP visits, specialist visits (with referral and private), and hospitalizations.

**Table 2:** Determinants of outpatient physician visits and hospitalizations.

Independent variables	Number of GP visits			Number of specialist visits with a referral			Number of specialist private visits			Number of hospitalizations		
	*B*	IRR (95% CI)	*p*‐value	*B*	IRR (95% CI)	*p*‐value	*B*	IRR (95% CI)	*p*‐value	*B*	IRR (95% CI)	*p*‐value
**Predisposing characteristics**												
Age (in years)	−0.01	1.00 (0.99–1.00)	0.059	−0.01	0.99 (0.99–1.00)	0.051	−0.01	**0.99** (0.98–1.00)	**0.025**	−0.01	0.99 (0.98–1.00)	0.099
Gender: Female (Ref.: Male)	0.08	1.09 (0.96–1.22)	1.179	0.03	1.03 (0.90– 1.19)	0.660	0.10	1.11 (0.91–1.35)	0.323	0.23	1.26 (0.91–1.73)	0.168
Family status: Never married (Ref.)	0^a^	1	—	0^a^	1	—	0^a^	1	—	0^a^	1	—
Married/Living with a partner	0.08	1.08 (0.91–1.28)	0.395	0.19	1.21 (0.99–1.49)	0.065	0.05	1.05 (0.81–1.35)	0.727	−0.21	0.81 (0.54–1.20)	0.291
Divorced/Widowed	0.15	1.16 (0.93–1.44)	0.194	0.24	1.27 (0.99–1.64)	0.061	0.07	1.07 (0.77–1.49)	0.684	−0.20	0.82 (0.50–1.37)	0.452
Family size	0.08	**1.08** (1.03–1.14)	**0.002**	0.01	1.01 (0.95–1.07)	0.873	0.01	1.01 (0.94–1.08)	0.818	0.04	1.04 (0.92–1.18)	0.547
Place of residence: Capital (Sofia) (Ref.)	0^a^	1	—	0^a^	1	—	0^a^	1	—	0^a^	1	—
Regional city	0.05	1.05 (0.87–1.27)	0.604	−0.04	0.97 (0.78–1.20)	0.752	−0.13	0.88 (0.68–1.14)	0.332	0.26	1.29 (0.80–2.10)	0.301
Small town	0.15	1.16 (0.93–1.44)	0.183	0.03	1.03 (0.80–1.32)	0.814	−0.17	0.84 (0.62–1.14)	0.270	0.65	**1.92** (1.13–3.25)	**0.016**
Rural area	0.06	1.06 (0.84–1.34)	0.631	0.02	1.02 (0.78–1.34)	0.862	−0.02	0.98 (0.70–1.38)	0.924	0.23	1.26 (0.68–2.34)	0.472
Education level (from level 0–2 to level 8)	−0.04	0.96 (0.91–1.02)	0.205	0.01	1.01 (0.95–1.08)	0.688	−0.09	**0.91** (0.84–1.00)	**0.042**	−0.06	0.94 (0.82–1.08)	0.393
Employment status: Full‐time job (Ref.)	0^a^	1	—	0^a^	1	—	0^a^	1	—	0^a^	1	—
Part‐time job/unemployed	0.03	1.03 (0.89–1.20)	0.678	0.06	1.06 (0.90–1.25)	0.496	−0.24	**0.79** (0.63–0.99)	**0.043**	−0.14	0.87 (0.60–1.24)	0.438
Retired (due to age or disability)	0.13	1.14 (0.96–1.34)	0.128	0.13	1.14 (0.95–1.36)	0.158	−0.17	0.85 (0.64–1.12)	0.250	0.09	1.10 (0.77–1.57)	0.614
Trust in provider (from 1 = not at all to 5 = very much)	0.07	**1.07** (1.03–1.12)	**0.002**	0.06	1.06 (1.00–1.13)	0.069	0.03	1.03 (0.95–1.12)	0.455	0.08	1.08 (0.98–1.20)	0.128
**Enabling resources**													
Household income	−0.10	**0.90** (0.86–0.95)	**< 0.001**	−0.07	**0.93** (0.88–0.99)	**0.017**	0.01	1.01 (0.94–1.09)	0.750	−0.11	0.89 (0.79–1.01)	0.064
Statutory health insurance: Yes, continuous (Ref.: No/with interruptions)	0.34	**1.40** (1.10–1.79)	**0.007**	0.25	1.28 (0.94–1.75)	0.118	0.12	1.13 (0.83–1.54)	0.456	−0.34	0.71 (0.42–1.23)	0.222
Voluntary health insurance: Yes (Ref.: No)	0.04	1.04 (0.93–1.16)	0.539	0.00	1.00 (0.88–1.14)	0.985	0.04	1.04 (0.89–1.22)	0.598	−0.14	0.87 (0.64–1.18)	0.370
Too expensive health service (Ref.: Not applicable)	0.06	1.06 (0.94–1.20)	0.329	0.01	1.01 (0.88–1.15)	0.898	−0.03	0.98 (0.82–1.16)	0.777	−0.04	0.96 (0.72–1.28)	0.763
Long waiting times/waiting list (Ref.: Not applicable)	0.05	1.05 (0.95–1.16)	0.380	0.09	1.10 (0.98–1.23)	0.117	0.24	**1.28** (1.10–1.48)	**0.001**	−0.17	0.84 (0.66–1.09)	0.187
No time (work and personal commitments) (Ref.: Not applicable)	0.06	1.06 (0.95–1.18)	0.308	−0.13	**0.88** (0.78–1.00)	**0.048**	−0.15	0.86 (0.74–1.01)	0.061	−0.15	0.86 (0.65–1.16)	0.328
Distance and/or transportation difficulties (Ref.: Not applicable)	−0.03	0.97 (0.85–1.11)	0.645	0.01	1.01 (0.87–1.17)	0.893	0.22	**1.25** (1.04–1.51)	**0.018**	−0.06	0.94 (0.69–1.28)	0.689
Lack of equipment and supplies at the medical facility (Ref.: Not applicable)	−0.16	**0.85** (0.74–0.98)	**0.020**	−0.08	0.93 (0.80–1.08)	0.324	−0.07	0.93 (0.78–1.12)	0.436	0.26	1.30 (0.95–1.79)	0.105
**Need factors**													
Self‐rated health (from 1 = very bad to 5 = very good)	−0.17	**0.85** (0.78–0.91)	**< 0.001**	−0.21	**0.81** (0.75–0.88)	**< 0.001**	−0.24	**0.79** (0.71–0.89)	**< 0.001**	−0.08	0.93 (0.78–1.11)	0.394
Chronic disease: Yes (Ref.: No)	0.11	**1.11** (1.00–1.24)	**0.049**	0.15	**1.16** (1.03–1.31)	**0.016**	−0.02	0.99 (0.84–1.16)	0.851	0.29	**1.33** (1.00–1.76)	**0.047**

*Note:* Bold values indicate statistically significant (*p* < 0.05). Results of multiple negative binomial regression analysis.

Abbreviations: *B*, regression coefficient; CI, confidence interval; GP, general practitioner; IRR, incidence rate ratio; Ref., reference group.

The number of *GP visits* was positively associated with family size (IRR: 1.08; 95% CI: 1.03–1.14), trust in provider (IRR: 1.07; 95% CI: 1.03–1.12), continuous SHI (IRR: 1.40; 95% CI: 1.10–1.79), and the presence of a chronic disease (IRR: 1.11; 95% CI: 1.00–1.24). On the other hand, the number of GP visits was negatively associated with household income (IRR: 0.90; 95% CI: 0.86–0.95), lack of equipment and supplies at the medical facility (IRR: 0.85; 95% CI: 0.74–0.98) and better self‐rated health (IRR: 0.85; 95% CI: 0.78–0.91).

Regarding the use of *specialist visits with referral*, the presence of one or more chronic conditions was significantly associated with more consultations (IRR: 1.16; 95% CI: 1.03–1.31) and higher income (IRR: 0.93; 95% CI: 0.88–0.99), lack of time (IRR: 0.88; 95% CI: 0.78–1.00) and better self‐rated health (IRR: 0.81; 95% CI: 0.75–0.88) were associated with fewer visits to specialists with a referral.

The number of *specialist private visits* was positively associated with some access barriers such as long waiting times/waiting lists (IRR: 1.28; 95% CI: 1.10–1.48) and distance or transportation difficulties (IRR: 1.25; 95% CI: 1.04–1.51), whereas it was negatively associated with age (IRR: 0.99; 95% CI: 0.98–1.00), education level (IRR: 0.91; 95% CI: 0.84–1.00), part‐time employment or unemployment (IRR: 0.79; 95% CI: 0.63–0.99) and better self‐rated health (IRR: 0.79; 95% CI: 0.71–0.89).

Respondents from small towns were *hospitalized* more frequently (IRR: 1.92; 95% CI: 1.13–3.25) than residents of the capital (reference group) and patients with chronic diseases were also admitted to the hospital more often (IRR: 1.33; 95% CI: 1.00–1.76).

## 4 Discussion

### 4.1 Implications of the Findings

The results of negative binomial regressions (with physician visits and hospitalizations) show that most of the predisposing, enabling, and need characteristics were associated with use of health care services. Nevertheless, outcome variables were not significantly associated with gender, family status, and VHI status.

With regard to *predisposing characteristics*, several individual factors were significantly associated with the outcomes. Although age is an indicator with a seemingly predictable influence on health care service use, our study do not find consistent association between these variables. Furthermore, the results of various studies show inconsistencies in the strength and direction of this association that differ substantially depending on other participant characteristics [2, 20, 21, 22]. Nonetheless, our results demonstrated a negative association between age and specialist private visits. There is room for additional research in this area, such as looking into the association between age and the presence of financial barriers. Other research investigating the determinants of health services utilization in the context of recession and during the later stages of the COVID‐19 pandemic also found a negative association between age and specialist visits [23, 24].

Former studies often suggest relationships between gender and utilization, with women having a higher number of examinations in most cases [16, 21, 25]. However, there are also studies that find lower utilization among female than men [14, 26] or others that find no significant association between gender and health care service use [4, 22].

Our findings for marital status revealed no independent influence on health care utilization, implying that the observed disparities are due to other individual characteristics.

Although household size is not a commonly observed determinant of use, other studies indicated that individuals with children in their own household had a higher number of specialist visits [23]. Our findings demonstrate that having more household members is associated with more GP visits, which we assume is due to mandatory preventive health check‐ups and increased attendance in childhood.

Our results suggested that people living in small towns have a higher average number of hospitalizations, which can be explained by the lack of specialist doctors in these settlements, which makes outpatient treatment more difficult. Hence, this sets the prerequisites for delays in treatment in outpatient care, which may lead to deteriorate health outcomes, out‐of‐pocket payments, unmet need, and increased hospitalizations for individuals living in smaller places of residence.

Our findings regarding the impact of the education level indicate that higher levels of education were associated with fewer number of private visits to a specialist. We can assume that higher education helps to solve the health problem more successfully, with fewer consultations. This suggestion is in line with Grossman's theory for the demand for health and healthcare, according to which education raises the marginal productivity of human capital and the production of health becomes more efficient, at lower costs [27]. A study in Ireland also found a negative association between education level and healthcare services utilization: those with lower levels of education had slightly more GP visits than those with higher levels of education [14]. However, in Greece, lower education was associated with lower utilization of outpatient services (GP and specialist consultations) [24].

Our results show a fewer specialist private visits for unemployed and part‐time employed individuals that could be explained by cost barriers, therefore, the association between unemployment and affordability of health services should be investigated further. Cost barriers could contribute to delay and underutilization of health services, deterioration of health, and consequently increased utilization and costs. Other studies demonstrated that not‐working individuals have a higher number of outpatient physician visits compared to those who are at work and some authors suggest that unemployment may contribute to poorer health [14, 24, 26]. The COVID‐19 pandemic has highlighted the role that trust plays in all aspects of health care delivery and health systems [28, 29]. Previous research demonstrated that patients with a lower level of trust in their physicians were more likely to report that requested or needed services were not provided, were less satisfied with their care, were less likely to follow the doctor's advice and were less likely to report symptom improvement [30]. Other study found that trust in experts and science affects attitudes and perceptions of misinformation and predicts susceptibility to false, unjustified, and biased claims [31]. Our findings on the influence of trust support the more widely held hypothesis that greater trust is associated with higher utilization, although studies in the field show that mistrust directs patients to other and additional services, increasing utilization and costs [32].

Policies aimed at improving physician‐patient relationships, enhancing communication, and ensuring that providers are perceived as trustworthy could encourage more consistent use of outpatient healthcare.

With regard to *enabling resources*, the higher number of GP and specialist visits in lower‐income groups can be explained by poorer health status and low financial barriers to service use. Furthermore, the cost barrier (“too expensive health service”) did not significantly influence the number of health care services used. These findings contradict previous study results, that demonstrated shift in outpatient care utilization toward higher income groups [33]. Therefore, we cannot rule out the existence of income barriers and social‐economic inequalities in the use of health services in Bulgaria.

Many studies have unequivocally demonstrated the favorable impact of health insurance on healthcare utilization. The findings also have shown that service utilization differs according to the kind of health insurance (public or private), its duration, and the degree of coverage [2, 5]. Our results confirmed the positive influence of SHI on the number of GP consultations and this is to be expected given the gate‐keeping role of GPs. Despite our anticipation that VHI would have a positive influence on the number of specialist private visits, no statistically significant association was found.

Other access barriers also showed a statistically significant impact on outpatient care utilization. The “lack of time due to other commitments” is related to fewer visits to a specialist with a referral and “long waiting times” and “distance problems” make respondents use paid examinations more often. Possible explanations are insufficient numbers of referrals leading to delays in examinations or a lack of GPs and specialists contracted by the NHIF in some remote areas [9]. Due to these problems of access to services under the SHI, patients have to seek alternatives such as private services payed out of pocket.

Investing in the public healthcare infrastructure can reduce the financial burden on individuals and ensure that healthcare is equitable. Expanding SHI coverage and increasing the availability of primary and specialized services in underserved areas would help ensure regular access to care, potentially preventing the need for more expensive, reactive treatments later on.

Finally, the vast majority of studies have identified *need factors* as strong predictors of health services utilization [2, 4, 5]. Poor self‐assessed health status and the presence of chronic disease were significant predictors of increased service use in most studies. Our analysis has shown that poor self‐rated health is associated with more GP visits, more visits to a specialist with a referral, and more private consultations. Having a long‐term (chronic) disease is a positive predictor for GP visits, visits to a specialist with a referral, and hospitalizations. This highlights the need for healthcare policies that focus on proactive care for chronic conditions, such as better disease management programs, patient education, and early intervention strategies.

We should notice that the number of physician visits and hospitalizations in the current study is higher compared to previous studies. A survey conducted in 2021 showed an average number of GP examinations of 2.93, compared with 4.14 examinations in our post‐pandemic study [33]. Furthermore, specialist examinations increased from 1.91 to over 2.51 and the average number of hospitalizations increased from 0.94 to 1.64. Increased utilization in the post‐pandemic period could be explained by delayed and postponed examinations during the pandemic, deteriorated health status as a result of untimely care, and recovery of activity levels after the lifting of any restrictive measures.

### 4.2 Limitations of the Study

This study has several limitations. The study period includes the last 2 months of the COVID‐19 epidemic emergency as well as the subsequent months and there were no formal restrictions or other counter‐epidemic measures affecting the provision and use of health services. However, it is possible that restrictions from the previous pandemic period had an impact that was not examined. Due to the survey method (online questionnaire and snowball sampling method), the sample was not representative of the general population. Self‐reported data are based on perception and may also lead to recall bias. The cross‐sectional study design investigates the determinants of health service utilization at particular time and context, without tracking their change over time. Possible interactions between factors were not explored.

## 5 Conclusions

This original application of the Andersen model in our country provides new insights into health service utilization and permits comparative analyses based on results in different countries and health systems. The findings of this study indicate increase in outpatient health care services use and hospitalizations and showed that need factors are the strongest driver of healthcare use. Additionally, our study demonstrated that predisposing characteristics and enabling resources are also important for the number of outpatient visits in Bulgaria in the post‐pandemic period. The flexibility of the model allows new determinants (e.g., vaccination status) to be added, enabling future research to focus on individuals at risk of underuse or overuse. The major challenge would be to distinguish between appropriate, over‐, or under‐utilization of health care services. We need further evidence on the effect of different levels of utilization on the population's health.

## Author Contributions

**Svetlana Panayotova:** data curation, formal analysis, investigation, resources, writing – original draft, writing – review and editing. **Elka Atanasova:** conceptualization, formal analysis, investigation, methodology, supervision, writing – original draft, writing – review and editing.

## Conflicts of Interest

The authors declare no conflicts of interest.

## Transparency Statement

The lead author Svetlana Panayotova affirms that this manuscript is an honest, accurate, and transparent account of the study being reported; that no important aspects of the study have been omitted; and that any discrepancies from the study as planned (and, if relevant, registered) have been explained.

## Data Availability

The data that support the findings of this study are available on request from the corresponding author. The data are not publicly available due to privacy or ethical restrictions. The corresponding author Svetlana Panayotova had full access to all of the data in this study and takes complete responsibility for the integrity of the data and the accuracy of the data analysis. Data available on request due to privacy/ethical restrictions.
